# Structure‒function‒pathogenicity analysis of C-terminal myocilin missense variants based on experiments and 3D models

**DOI:** 10.3389/fgene.2022.1019208

**Published:** 2022-10-04

**Authors:** Biting Zhou, Xiaojia Lin, Zhong Li, Yihua Yao, Juhua Yang, Yihua Zhu

**Affiliations:** ^1^ Department of Ophthalmology, The First Affiliated Hospital of Fujian Medical University, Fuzhou, China; ^2^ Department of Bioengineering and Biopharmaceutics, School of Pharmacy, Fujian Medical University, Fuzhou, China

**Keywords:** MYOC, primary open-angle glaucoma, structure, function, pathogenicity

## Abstract

MYOC is a common pathogenic gene for primary open-angle glaucoma and encodes the protein named myocilin. Multiple MYOC variations have been found, with different clinical significance. However, the pathogenesis of glaucoma induced by MYOC mutations has not been fully clarified. Here, we analyze the molecular and cellular biological differences caused by multiple variant myocilins, including protein secretion characteristics, structural changes, subcellular localization, cellular autophagic activity and oxidative stress. Denaturing and nondenaturing electrophoresis showed myocilin to be a secreted protein with the tendency to self-oligomerize. The full-length myocilin and its C-terminal cleavage fragment are secreted. Secretion analysis of 23 variant myocilins indicated that secretion defects are closely related to the pathogenicity of MYOC variants. Structural analysis showed that the alteration of steric clash is associated with the secretion characteristics and pathogenicity of myocilin variants. Immunocytochemistry results demonstrated that mutated myocilins are retained in the endoplasmic reticulum and disrupt autophagy. MTT assay, MitoTracker staining, and DCFH-DA staining showed increased oxidative injury in cells expressing MYOC mutants. Taken together, MYOC mutations are able to induce cell dysfunction via secretion defects and intracellular accumulation resulting from steric clash alterations.

## Introduction

Primary open-angle glaucoma (POAG) is the most common type of glaucoma, with an overall global prevalence of 2.4% (Zhang et al., 2021). The prevalence of POAG varies among different countries, races, sexes and ages. The highest prevalence is in the African population, at 4.5% (Kapetanakis et al., 2016). The prevalence of POAG in Europe and Asia is 2.1 and 1.9% (Kapetanakis et al., 2016), respectively. Due to the large-scale population and the rapid expansion of population aging in Asia, it is estimated that the prevalence of POAG in Asia will increase to 49% by 2050 (Kapetanakis et al., 2016). People with POAG experience high intraocular pressure (IOP) and progressive optic nerve degeneration (Lei et al., 2019), rendering POAG a main cause of blindness and a severe public health problem. High IOP, as a result of damage to the trabecular meshwork (TM) and increased outflow resistance, is a major but the only controllable risk factor for POAG.

It has been reported that the risk of POAG among first-degree family members of POAG patients is significantly higher than that among first-degree family members of non-POAG patients, indicating that POAG has genetic susceptibility (Gong et al., 2007). MYOC is a major pathogenic gene of POAG, with a mutation frequency of 10%–30% (Huang et al., 2018). MYOC mutations are responsible for 2%–4% of POAG cases and 8%–36% of juvenile open-angle glaucoma (JOAG) cases (Wiggs et al., 1998; Fingert et al., 1999; Souzeau et al., 2013). To date, more than 270 MYOC variants have been found, including POAG-causing mutations, neutral polymorphisms and variations with uncertain clinical significance (Hewitt et al., 2008).

MYOC encodes myocilin, a secreted and glycosylated protein that is expressed in ocular and nonocular organs such as the heart and skeletal muscle. Nevertheless, mutation of MYOC only leads to glaucoma lesions in eyes. MYOC consists of three exons, and the olfactomedin (OLF) in the C-terminal of myocilin is estimated to houses over 90% of reported POAG-causing mutations (Scelsi et al., 2021). The exact function of myocilin remains unclear. It was reported that myocilin may be involved in interaction between cells and the extracellular matrix (ECM) (Kasetti et al., 2016; Joe et al., 2017), neurite outgrowth (Jurynec et al., 2003), cell migration (Kwon and Tomarev, 2011), mitochondrial injury of TM cells (Sakai et al., 2007), oligodendrocyte differentiation and myelination of the optic nerve (Kwon et al., 2014) and programmed cell death during retinal development (Koch et al., 2014). Previous studies have shown that MYOC knock-in or knockout mice exhibit no POAG phenotype, supporting a gain-of-function disease model (Kim et al., 2001; Gould et al., 2004; Scelsi et al., 2021). Furthermore, different MYOC mutants have been reported to have variable effects on cellular biological functions. However, functional studies reported thus far are not all-inclusive; namely, the secretion property, cellular stress responses, ECM production, cell proliferation and adhesion of mutant myocilins have not been studied together (Gobeil et al., 2006; Jia et al., 2009; Stothert et al., 2014; Yan et al., 2020), thus creating a gap in the understanding of the precise molecular mechanism that leads to POAG for each mutation. This wide functional heterogeneity of MYOC missense mutations calls for a structure‒function correlation approach.

Therefore, the purpose of this study was to decipher the factors that determine the pathogenicity of MYOC variations in TM. To accomplish this, we examined molecular and cellular biological differences mediated by multiple MYOC variants, including protein secretion characteristics, structural changes, subcellular localization, cellular autophagy activity and oxidative stress.

## Materials and methods

### Myocilin constructs

cDNAs encoding wild-type myocilin (MYOC, NM_000261.1) and mutated myocilins were cloned into the *EcoRI-NheI* sites of the pcDNA3.1 mammalian expression vector. The primer sequences used to generate these myocilin cDNAs are shown in Supplementary Table S1. A total of 23 MYOC mutant plasmids were constructed. All constructs used in this study were verified by direct DNA sequencing.

### Cell culture and transfection

Details of the cells used in this study are shown in the table below.

**Table:** 

Name	Source
Human embryonic kidney 293T (HEK 293T) cells	GENE (Shanghai, China)
COS-7 cells	Beyotime (Shanghai, China)
Immortalized human trabecular meshwork cells (iHTMCs)	Meisen CTCC (Zhejiang, China)

HEK 293T and COS-7 cell lines were maintained in Dulbecco’s modified Eagle’s medium (DMEM) supplemented with 10% fetal bovine serum (FBS), 100 U/ml penicillin and 100 µg/ml streptomycin. iHTMCs were cultured in DMEM/F-12 medium supplemented with 15% FBS, 100 U/ml penicillin and 100 µg/ml streptomycin. All cultures were maintained at 37°C in a humidified atmosphere of 95% air and 5% CO_2_. Cells were transfected with plasmids containing wild-type (WT) or mutant MYOC cDNA by transfection reagents (Lipofectamine 2000, Invitrogen, CA, United States, Cat# 11668-019 or X-tremeGene™ 360 Transfection Reagent, Roche, Mannheim, Germany, Cat# 8724105001) following the manufacturer’s instructions. To ensure the stability and consistency of the transfection efficiency of various plasmids, cells were simultaneously transfected with a cDNA construct encoding GFP and the transfection rate was measured *via* calculating the ratio of GFP-positive cells under a fluorescence microscope.

### Cellular fractions preparation

After 48 h of transfection with OPTI-MEM (no FBS), the culture medium was harvested and centrifuged at 5,000 × g for 5 min at 4°C to remove dead cells, followed by 16,000 × g for 10 min at 4°C for further removal of cellular debris. The collected culture medium was concentrated using Amicon Ultra15 Centrifugal Filters (10K, Millipore, MA, United States, Cat# UFC801008) by centrifugation at 4,500 × g for 30 min at 4°C. Adhered cells were washed twice with ice-cold PBS, followed by resuspension in RIPA cell lysis buffer (Beyotime, Cat# P0013B) containing the proteinase inhibitor PMSF (1:100, Beyotime, Cat# ST505) and the phosphatase inhibitor PhosSTOP (Roche, Mannheim, Germany, REF 04906845001). After incubation on ice for 30 min and centrifugation, the supernatants (soluble cell fraction) were carefully separated from the pellets (insoluble cell fraction). For denaturing SDS‒PAGE, aliquots of culture medium and cell fractions were treated with 4× protein loading buffer containing β-mercaptoethanol and SDS and boiled for 10 min. For nondenaturing PAGE, samples were treated with 4× protein loading buffer without β-mercaptoethanol, SDS and boiling. Samples were normalized for protein content using the Bradford assay using bovine serum albumin as a control.

### Western blotting and antibodies

For western blotting analysis, aliquots of culture medium and intracellular fractions of cultured cell lines (both soluble and insoluble) were fractionated by 10%–12% SDS‒PAGE. For non-denaturing PAGE, samples were loaded onto 4%–12% polyacrylamide gradient gels (GeneScript, Nanjing, China, Cat# M00654) without SDS. The proteins were transferred onto a PVDF membrane and blocked for 1 h at room temperature with 5% milk. The membrane was incubated with primary antibodies against different domains of myocilin: anti-myocilin from Millipore (1:2,000, CA, United States, Cat# MABN866) corresponding to the N-terminal fragment (aa 33-214) of human myocilin and MYOC rabbit polyclonal antibody derived from Abclonal (1:2,000, Wuhan, China, Cat# A1589) corresponding to the C-terminal fragment (aa 245-504). After overnight incubation at 4°C, the membrane was incubated with the corresponding horseradish peroxidase (HRP)-conjugated goat anti-mouse (1:5,000, Affinity Biosciences, Cat# S0002) or rabbit (1:5,000; Affinity Biosciences, Cat# S0001) secondary antibody at room temperature for 2 h. Enhanced chemiluminescence was used to visualize protein bands.

### Immunocytochemistry assay

Cells were grown on confocal plates, fixed and permeabilized with methanol for 15 min at −20°C, followed by three washes with PBS. The cells were incubated for 1 h in blocking buffer (5% normal donkey serum and 0.3% Triton X-100 in PBS), followed by incubation with an anti-MYOC primary (1:400, Millipore), anti-Grp94 primary (1:200, Affinity Biosciences, Cat# AF5287), or anti-LC3 primary (1:200, Cell Signaling Technology, Cat# 4108) antibody overnight at 4°C. After gentle washing, the respective fluorescent secondary antibody purchased from Abcam (donkey anti-mouse IgG H&L, Alexa Fluor 594, Cat#ab150108, 1:1,000; donkey anti-rabbit IgG H&L, Alexa Fluor 488, Cat#ab150073, 1:1,000) was added, and the cells were incubated at 37°C for 2 h in the dark. Nuclei were stained with 0.01 mg/ml Hoechst 33342. At least six fields of view were photographed for each group of cells using a laser-scanning confocal microscope (Leica, Nussloch, Germany) and a digital camera using LAS AF software.

### Cell viability assay

Cell viability was detected using 3-(4,5-dimethylthiazol-2-yl)-2,5-diphenyltetrazolium bromide (MTT; Yeasen, Shanghai, China, Cat# 40201ES80). Cells were seeded in 96-well plates. After appropriate treatment, the cells were incubated in culture medium with 0.5 mg/ml MTT for 4 h. The formed dark blue crystals were dissolved with DMSO, and absorbance was measured at 570 nm using a microplate reader.

### Measurement of reactive oxygen species and mitochondrial membrane potential

Cellular oxidative injury was evaluated by detecting reactive oxygen species (ROS) generation and mitochondrial membrane potential (MMP). The generation of ROS was measured by the DCFH-DA probe (Beyotime, Cat#S0033S), and MMP was detected by the Mito-Tracker Red CMXRos probe (Beyotime, Cat#C1049) according to the manufacturers’ instruction and previous studies (Jurado-Campos et al., 2021; Gu et al., 2022). Briefly, cells were cultured and treated in 24-well plates, and incubated with corresponding probes at 37°C in the dark for 30 min. After washing with PBS, the samples were examined under a fluorescence microscope.

### Structural analysis

Swiss-PdbViewer software v4.1.0 was employed for structural analyses. The crystal structure of human myocilin-OLF (PDB: 4 WXQ) was selected as a template. Mutations were induced in the template one at a time using the mutate tool of the Swiss-PdbViewer software, and analyses were performed based on the “best” rotamer of the new amino acid. We examined the 1) solvent accessibility of the residues, 2) gain/loss of H-bonds, 3) induction of steric clash, 4) change in surface electrostatic potential (SEP) and 5) alteration of the molecular surface.

### Statistical analysis

All experiments were performed at least 3 times. Data are presented as the mean ± SD. GraphPad Prism eight was used to determine statistical significance. Data were analyzed using one-way ANOVA with Tukey’s post-hoc test for comparisons between more than two groups. *p* < 0.05 was considered statistically significant.

## Results

### Molecular characteristics of myocilin

**FIGURE 1:**
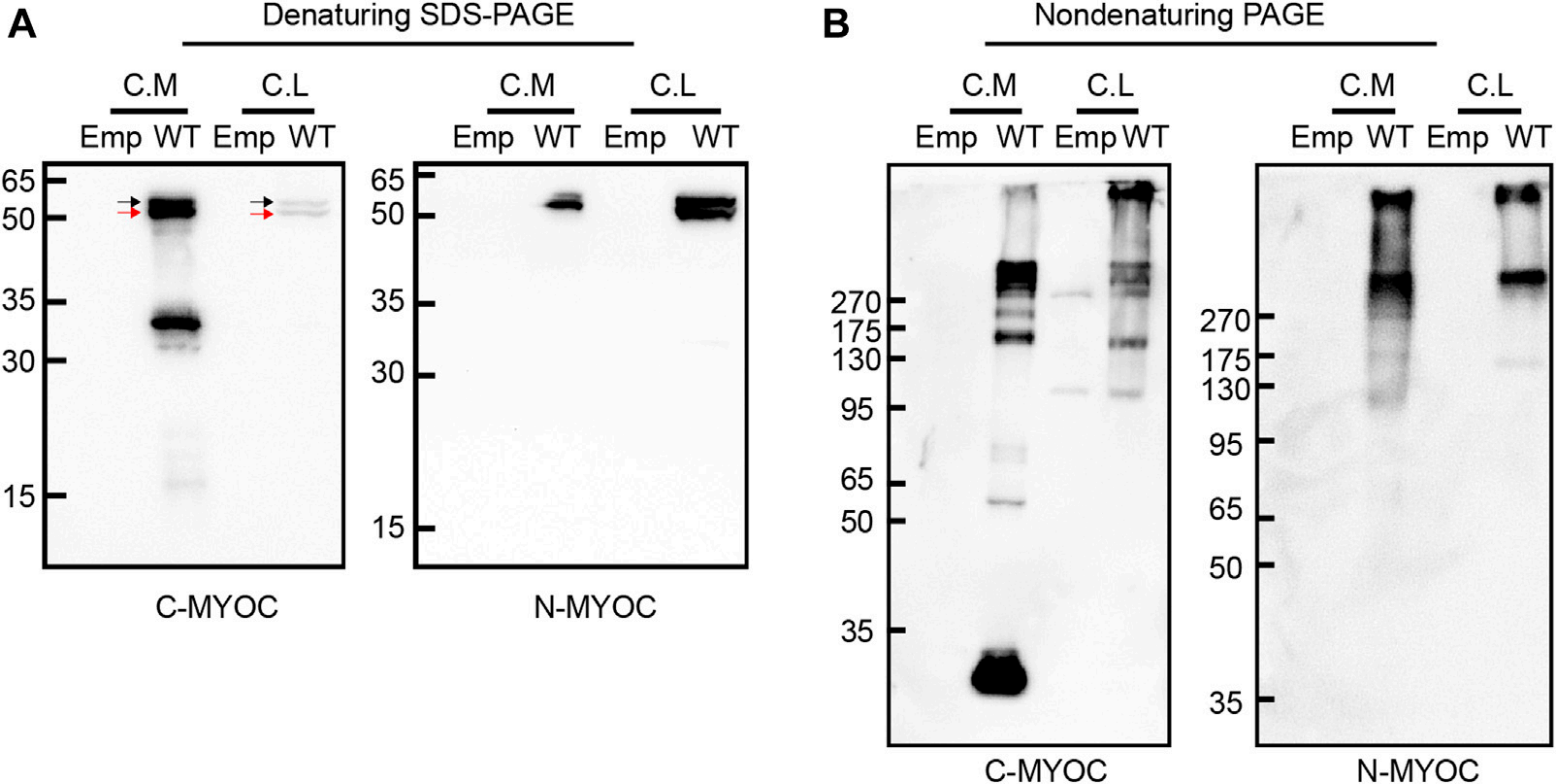
Molecular characterization of intracellular and extracellular myocilin. **(A,B)** A cDNA construct encoding the empty vector (Emp) or wild-type (WT) myocilin was transfected into HEK 293T cells. Forty-eight hours after transfection, myocilin was analyzed in the culture medium (C. M) and cell lysates (C. L) by denaturing SDS–PAGE **(A)** and nondenaturing PAGE **(B)**. Myocilin detection was carried out by western blotting using an anti-C-terminal (C-MYOC) or anti-N-terminal (N-MYOC) MYOC antibody. Black arrow: glycosylated myocilin band. Red arrow: nonglycosylated myocilin band. C. M, culture medium; C. S, RIPA-soluble cell fraction; C. I, RIPA-insoluble cell fraction.

We overexpressed WT myocilin in HEK 293T cells and detected the molecular characteristics of intracellular and extracellular myocilin via denaturing SDS–PAGE and nondenaturing PAGE. As shown in Figure 1A, myocilin was present in the cell medium under denaturing conditions, with two bands near 50–65 kDa that were recognized by two different myocilin antibodies. The upper band was concluded to be glycosylated myocilin (black arrow) and the lower band to be nonglycosylated myocilin (red arrow) (Caballero and Borrás, 2001). There was also a band near 30–35 kDa, which was detected only when using a C-terminal antibody; it was concluded to be the C-terminal cleavage portion of myocilin. In addition, intracellular myocilin presented as a doublet band slightly larger than 50 kDa. Nondenaturing PAGE was used to observe the native status of proteins, which keep higher-order structure without treatment of SDS, DTT and boiling. Under nondenaturing conditions (Figure 1B), except for a band smaller than 35 kDa in cell medium, bands indicating multiple oligomeric states with molecular weights of over 130 kDa were observed for both intracellular and extracellular myocilin, supporting a tendency of myocilin to self-oligomerize.

### Secretion profile of myocilin variants

**FIGURE 2:**
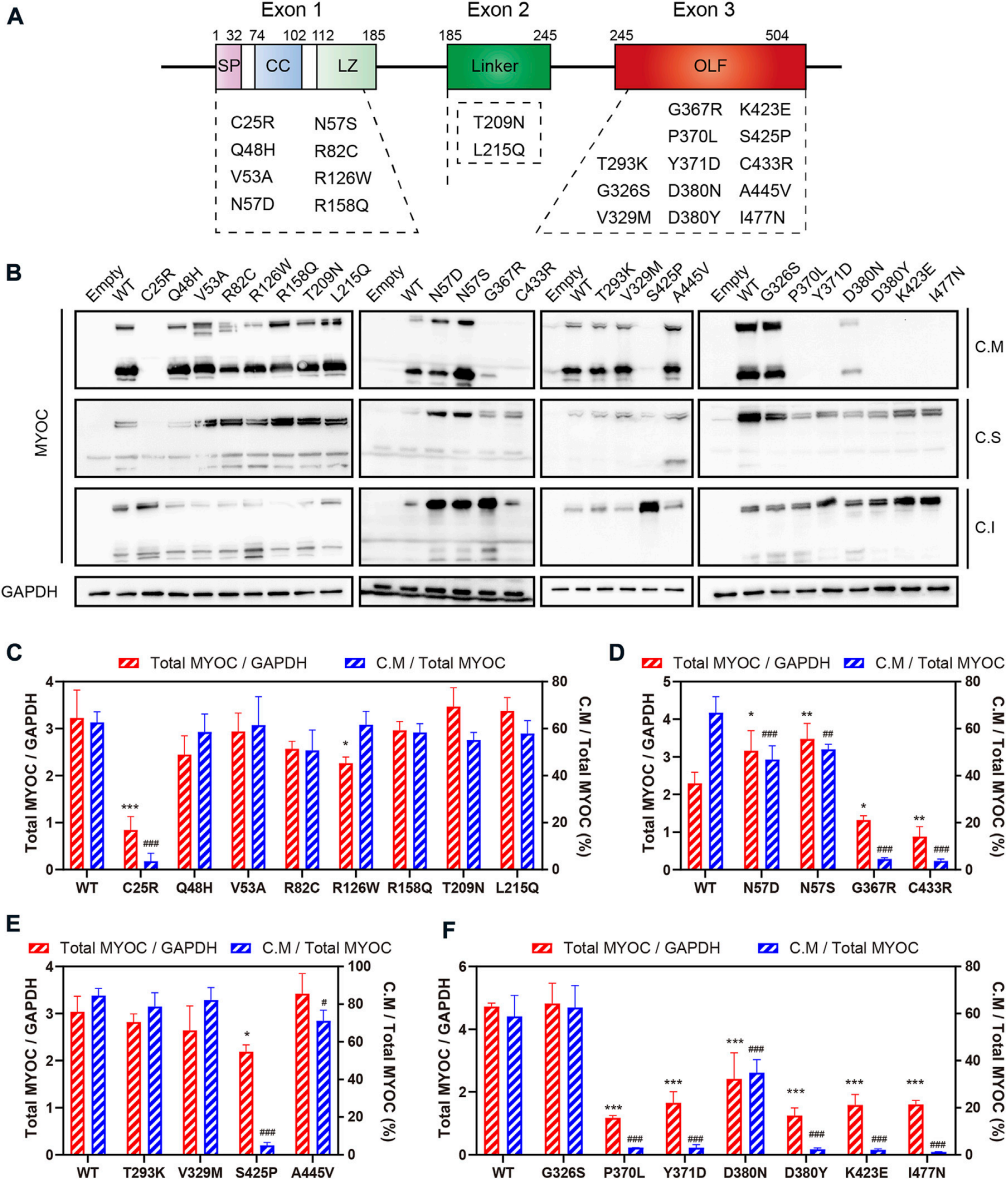
Secretion analysis of myocilin variants. **(A)** Structure of myocilin. MYOC gene has three exons, encoding myocilin that consists of signal peptide (SP), coiled-coil domain (CC), leucine zipper (LZ), linker and olfactomedin (OLF) domain. The variants listed in the dotted box are the variants studied in this study. **(B)** Western blot analysis of intracellular and extracellular myocilin in HEK 293T cells expressing different MYOC variants. Forty-eight hours after transfection, myocilin was detected in the culture medium (C. M) and in RIPA-soluble and -insoluble cell fractions (C. S and C. I) by denaturing SDS‒PAGE. An MYOC antibody that recognizes the C-terminus of myocilin was used to detect expression of myocilin. **(C–F)** Densitometric analysis of immunoblotting bands. C.M = full myocilin detected in C.M + short C-terminal products detected in C.M. Total MYOC = C.M + C.S + C.I. N = 3.

Myocilin contains three exons, and the N- and C-termini are the two major regions of homology. To investigate the effect of variations in different domains on myocilin secretion, we constructed 23 MYOC plasmids with variations covering all three exons (Figure 2A). The variants were chosen based on the structural and functional characteristics of myocilin (Supplementary Table S2), and all variants tested have been reported in previous human populations or pedigree screens for MYOC (Table 1). Among the 23 variants, ten have been described as glaucoma-causing mutations, ten are neutral polymorphisms, and three are considered uncertain.

**TABLE 1:** Localization, pathogenicity and secretion characteristics of 23 MYOC variants.

Number	Mutation	Location	Domain	Glaucoma causing	Secretion
1	C25R	Exon 1	SP	Uncertain pathogenicity	-
2	Q48H	Exon 1	CC	Neutral polymorphism	+
3	V53A	Exon 1	CC	Neutral polymorphism	+
4	N57D	Exon 1	CC	Neutral polymorphism	+
5	N57S	Exon 1	CC	Neutral polymorphism	+
6	R82C	Exon 1	CC	Neutral polymorphism	+
7	R126W	Exon 1	LZ	Neutral polymorphism	+
8	R158Q	Exon 1	LZ	Neutral polymorphism	+
9	T209N	Exon 2	Linker	Uncertain pathogenicity	+
10	L215Q	Exon 2	Linker	Glaucoma-causing mutation	+
11	T293K	Exon 3	OLF	Neutral polymorphism	+
12	G326S	Exon 3	OLF	Glaucoma-causing mutation	+
13	V329M	Exon 3	OLF	Neutral polymorphism	+
14	G367R	Exon 3	OLF	Glaucoma-causing mutation	-
15	P370L	Exon 3	OLF	Glaucoma-causing mutation	-
16	Y371D	Exon 3	OLF	Glaucoma-causing mutation	-
17	D380N	Exon 3	OLF	Uncertain pathogenicity	+
18	D380Y	Exon 3	OLF	Glaucoma-causing mutation	-
19	K423E	Exon 3	OLF	Glaucoma-causing mutation	-
20	S425P	Exon 3	OLF	Glaucoma-causing mutation	-
21	C433R	Exon 3	OLF	Glaucoma-causing mutation	-
22	A445V	Exon 3	OLF	Neutral polymorphism	+
23	I477N	Exon 3	OLF	Glaucoma-causing mutation	-

Note: SP, signal peptide; CC, coiled-coil domain; LZ, leucine zipper; OLF, olfactomedin domain. +: secretion, -: nonsecretion. The clinical significance of MYOC, variants refers to the study of Hewitt et al. (2008).

The plasmids constructed were transfected into HEK 293T cells, and proteins in the cell medium, soluble cell fraction and insoluble cell fraction were extracted and analyzed by denaturing SDS–PAGE using an antibody against the C-terminal region of myocilin. As shown in Figure 2B, among the N-terminal (exon 1 and exon 2) variants, only the C25R variant was absent from the culture medium, indicating that this variant was not secreted into the medium. In addition, five of the thirteen variants occurring in the C-terminus (exon 3) were secreted into the culture medium. Of the 14 secreted variants, ten featured neutral polymorphisms and two disease-causing mutations; the other two were defined as uncertain. Notably, 100% of the neutral-polymorphism proteins (10/10) were secreted, whereas 80% of the proteins encoded by disease-causing variants (8/10) were retained inside the cells (Table 1), suggesting that secretion is an important parameter that determines the pathogenicity of MYOC variations.

To further explore the effect of MYOC variations on the expression and secretion of myocilin, densitometric analysis was performed to quantify the level of myocilin in different cellular fractions (Figures 2C–F). We found that MYOC mutations located in C-terminus resulted in significantly decreased expression of total myocilin, which is mainly existed in insoluble cellular fraction (Figures 2D–F and Supplementary Figure S1). Interestingly, although D380N variation reduced the expression and secretion of myocilin, myocilin was still detectable in the culture medium (Figure 2F). Among the variations in N-terminus, C25R variation decreased the level of total myocilin dramatically and the protein was mainly existed in the insoluble fraction, which may be responsible for its nonsecretion (Figure 2C). Notably, variations in glycosylated site (N57) increased the level of total myocilin and decreased the percentage of C.M-myocilin (Figure 2D).

### Structural analysis of myocilin missense variations

**FIGURE 3:**
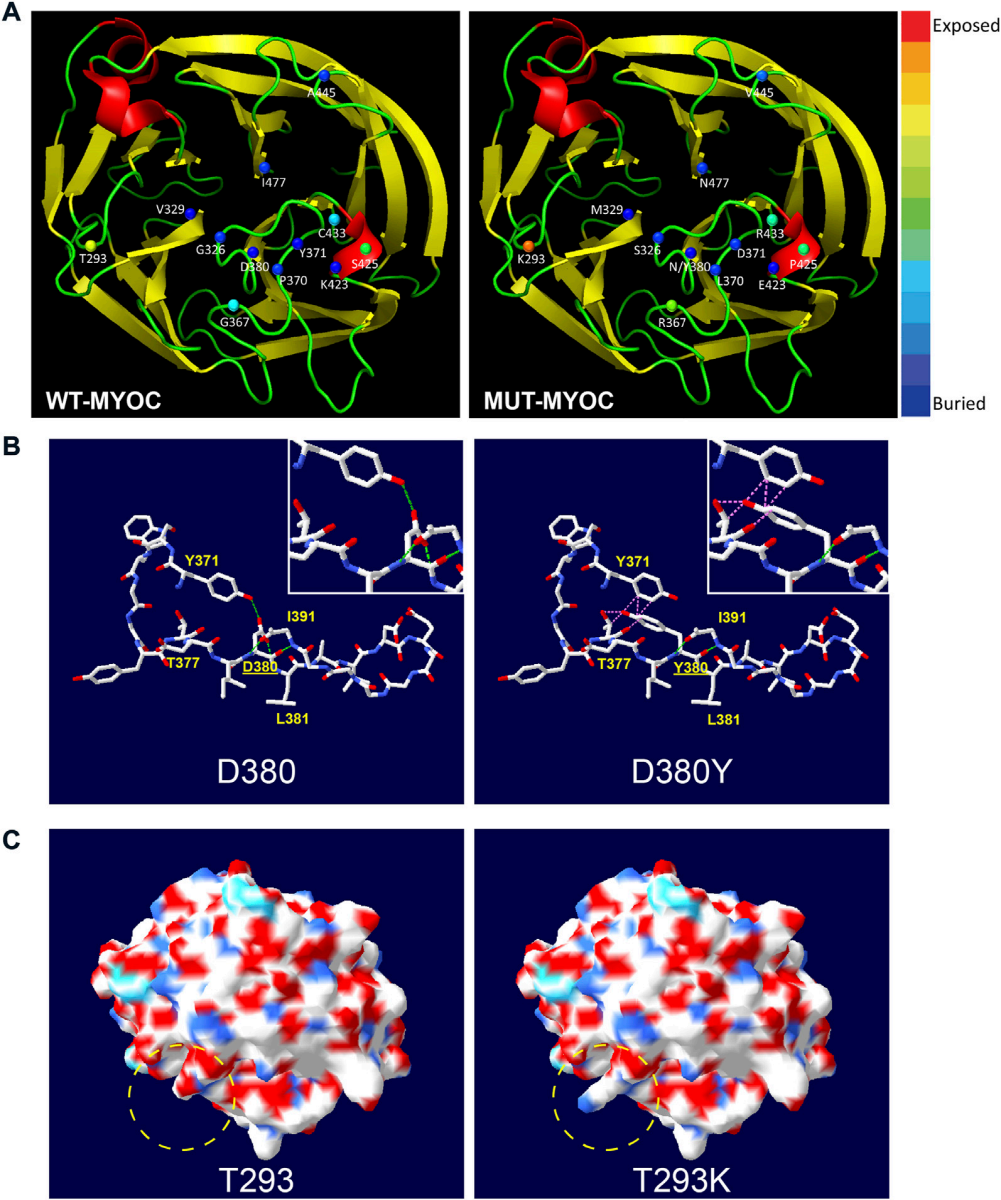
Structural alteration of myocilin-OLF induced by C-terminal MYOC variations using Swiss PdB Viewer. **(A)** Solvent accessibility analysis of WT-myocilin (left panel) and mutated-myocilin (right panel). A cartoon view of the myocilin-C-terminus homology (PDB: 4WXQ) model is shown. Twelve residues involved in 13 missense changes are represented as spheres colored according to solvent accessibility (using Swiss PdB Viewer, by which blue through red correspond to buried-through-exposed residues; see color bar). **(B)** Alteration of H-bonds and steric clashes induced by MYOC/p.D380Y mutation. H-bonds are drawn as green dotted lines, and clashes appear as pink dotted lines. **(C)** Changes in molecular surface including electrostatic potential (SEP) and structure of MYOC/p.T293K mutation. The molecular surface is colored according to SEP using Swiss PdB Viewer, with red‒white-blue corresponding to acidic-neutral-basic potential. The yellow dotted circle represents the region of significant alteration of SEP and surface structure. See also Supplementary Figures S2,S3.

From the perspective of protein structure, we assessed the structural alterations induced by the 13 variations in the OLF domain because most variations of nonsecreted myocilin and most pathogenic mutations occur in this region. The 3D model of OLF structure of myocilin (PDB: 4WXQ) was applied for structural analysis. As illustrated in Figure 3A, 6 of the 13 variations involve structurally exposed residues, including three POAG mutations, 2 neutral polymorphisms and one uncertain variation. S425P is a relatively buried residue, and another 6 variations did not change solvent accessibility. Next, we analyzed alterations in H-bonds and the induction of steric clash caused by MYOC variations. Figures 3B,C shows the common structural alterations in two MYOC mutants. As shown in Figure 3B, D380Y mutant results in loss of H-bond with L381 and Y371 and causes induction of steric clash with Y371 and T377. While Figure 3C shows that T293K mutant alters SEP from acidic to basic, and leads to the change of molecular surface. Structural alterations of other C-terminal variants are shown in Supplementary Figures S2,S3. Of the 13 variations, 9 alter the H-bonding pattern of the molecule (causing gain and/or loss of H-bonds with other residues), and three induce a change in steric clash. Furthermore, molecular surface analysis of characteristics including the SEP and surface structure revealed that alterations in both occur with the T293K, G367R, P370L, C433R and A445V mutations but that no SEP or surface structure alterations occur for G326S, V329M, Y371D, D380N, D380Y, K423E and I477N. Interestingly, S425P does not alter surface structure but does reduce acidic potential.

### Correlation analysis of structure-secretion-pathogenicity in myocilin variants

To research correlation among the structure, secretion and pathogenicity of myocilin variations, we analyzed another 20 C-terminal variations with secretion phenotypes determined by previous studies (Fan et al., 2006; Gobeil et al., 2006; Hogewind et al., 2007). The structural alterations and secretion status of a total of 33 C-terminal variations are summarized in Supplementary Table S3. An interesting relationship between secretion and alteration of steric clash was found. Among 10 variant myocilins with changes in steric clash, 100% present secretion defects; 90% are defined as mutation and 10% as uncertain. In addition, among 10 secreted myocilin variants, 100% show no change in steric clash, 20% are identified as mutation, 60% are considered nonpathogenic variations, and 20% are uncertain variations. Therefore, structural alteration (steric clash) may play important roles in influencing the secretion characteristics of myocilin variants, which determine the pathogenicity of myocilin variants.

### MYOC mutation induces retention of myocilin in the ER and impairs autophagic activity

**FIGURE 4:**
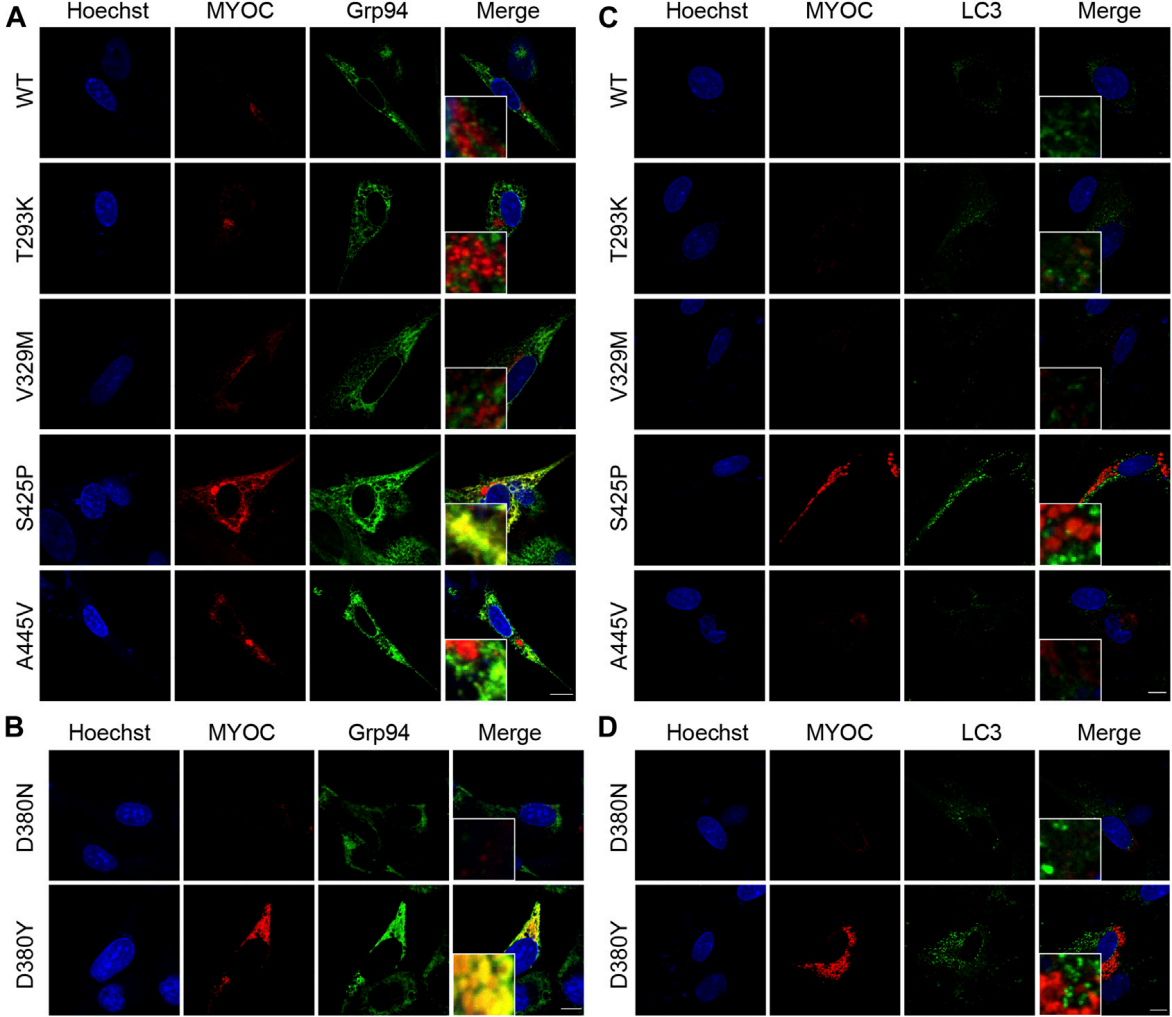
Nonsecreted MYOC mutants induce ER stress and impair autophagy. **(A,B)** Confocal double immunofluorescence for myocilin (red) and Grp94 (green) in iHTMCs transiently expressing WT or mutated MYOC. Nonsecreted mutations, including S425P and D380Y, show considerable colocalization of myocilin with Grp94. Scale bar: 25 μm. **(C,D)** iHTMCs expressing WT or mutant myocilins were immunolabeled with LC3 (green) and MYOC (red). S425P and D380Y mutants present increased intracellular myocilin and LC3, with no colocalization of these two proteins. Scale bar: 25 μm.

It was described in Donegan’s work that T293K, V329M, S425P and A445V variants were predicted with different clinical signification from original assignment. Therefore, we investigated the colocalization of myocilin and the ER marker Grp94 by confocal double immunofluorescence in iHTMCs expressing these variants, together with D380N and D380Y variants, which present opposite secretion property in the same site. As depicted in Figure 4A, few myocilin puncta were observed in cells transfected with WT or the T293K, V329M, or A445V-MYOC plasmid. In addition, no colocalization of myocilin and Grp94 was found in these cells. Conversely, iHTMCs expressing S425P-myocilin presented clustered myocilin puncta that prominently colocalized with Grp94. Furthermore, iHTMCs transfected with plasmids containing the D380N or D380Y MYOC variation shared the same location but exhibited opposite secretion phenotypes. We observed considerable codistribution of MYOC and Grp94 in cells expressing D380Y-myocilin compared with cells expressing D380N-myocilin (Figure 4B), suggesting that MYOC mutation induces retention of myocilin in the ER, which is likely to trigger ER stress.

The autophagy activity in cells expressing pathogenic or nonpathogenic myocilin variants was further explored to study the effect of MYOC mutation on cellular pathophysiology. As shown in Figure 4C, expression of LC3, an autophagic marker, was higher in iHTMCs expressing the S425P mutant than in iHTMCs expressing the WT protein or other variants. However, little or no colocalization of LC3 with myocilin was found at high magnification. Similarly, the D380Y mutation resulted in more MYOC and LC3 puncta than the D380N variation, but no colocalizing puncta was found (Figure 4D). Therefore, MYOC mutations may impair autophagy activity.

### MYOC mutations promote cellular oxidative stress

**FIGURE 5:**
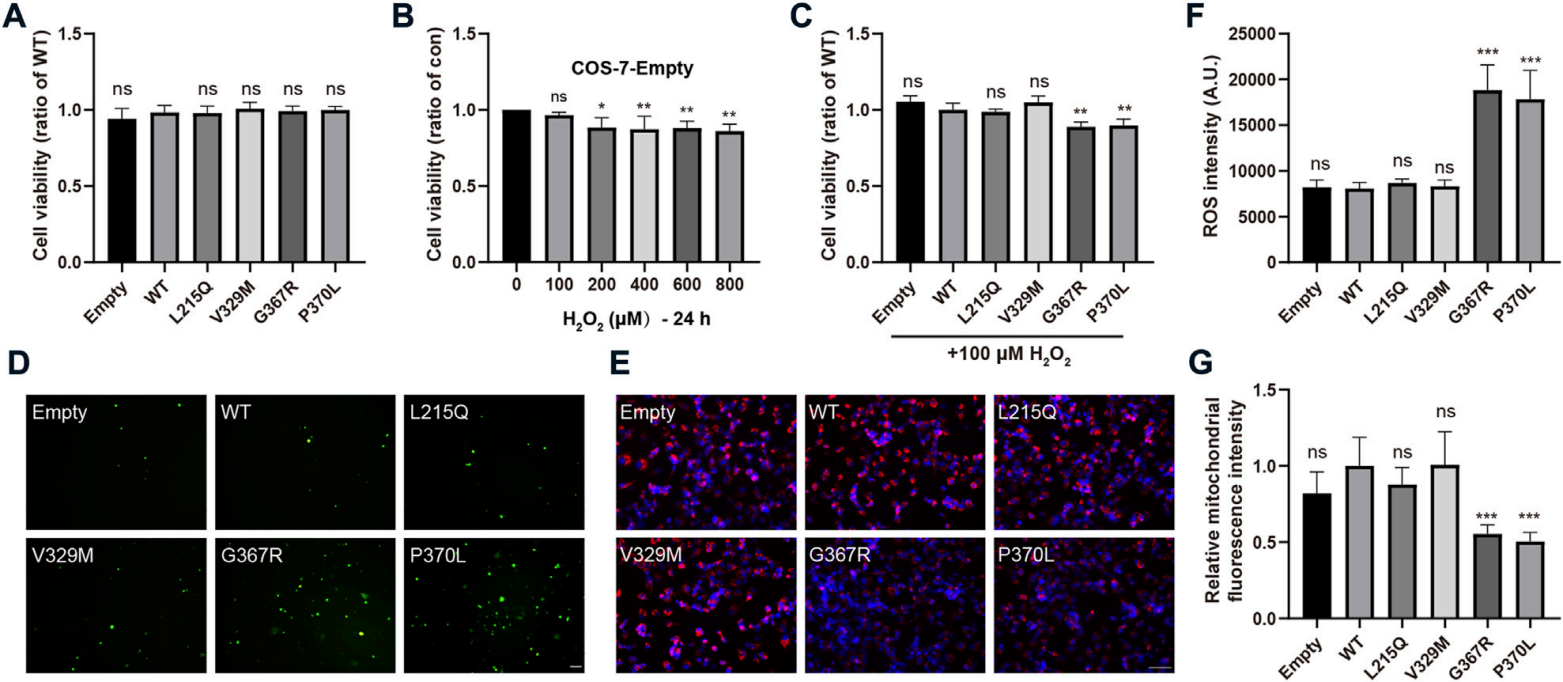
MYOC mutations induce cellular oxidative injury. **(A–C)** COS-7 cell viability was measured by MTT assays. **(A)** Expression of WT- or mut-myocilin had no effect on the viability of COS-7 cells. **(B)** Twenty-four hours of incubation with H_2_O_2_ at 100 μM did not inhibit the growth of COS-7 cells transduced with empty vector. **(C)** COS-7 cells transfected with different vectors were exposed to 100 μM H_2_O_2_ for 24 h. MYOC mutations increased the toxicity of H_2_O_2_ to COS-7 cells. **(D,F)** Increased ROS generation measured by DCFH-DA staining in COS-7 cells expressing G367R- or P370L-mutated myocilin. Scale bar: 100 μm. **(E,G)** Mitochondrial staining by MitoTracker showed that transfection of G367R- or P370L-mutated myocilin in COS-7 cells induced a decline in MMP. Scale bar: 100 μm. N ≥ 3. *p < 0.05, **p < 0.01, ***p < 0.001, ns: no significance.

To evaluate whether pathogenic MYOC mutations cause cellular oxidative stress, we transfected COS-7 cells with two secreted variants (one pathogenic variation L215Q and one neutral polymorphism V329M) and two nonsecreted mutants (G367R and P370L that were reported to cause severe POAG phenotypes), and H_2_O_2_ sensitivity, ROS generation and mitochondrial function were analyzed. Under physiological conditions, there were no significant differences in cell viability between cells transfected with WT MYOC and those transfected with MYOC variants (Figure 5A). Next, we demonstrated that treatment of control COS-7 cells with H_2_O_2_ did not decrease cell viability at a concentration of H_2_O_2_ below 100 μM (Figure 5B). When cells expressing WT MYOC were treated with 100 μM H_2_O_2_, no significant decrease in cell viability was observed. However, similar treatment of cells expressing G367R- or P370L-mutated myocilin significantly reduced cell viability, indicating higher sensitivity to H_2_O_2_ for mutant-expressing cells than WT protein-expressing cells (Figure 5C). ROS are the major product of oxidative stress and are mainly derived from mitochondria. Our assessment of ROS generation with the probe DCFH-DA and of mitochondrial function by MitoTracker staining suggested that nonsecreted MYOC mutants induce ROS accumulation and mitochondrial injury (Figures 5D–G). Conversely, cells expressing L215Q or V329M variant showed no significant difference in cell viability (under H_2_O_2_ treatment), ROS generation and mitochondrial function, compared to that in WT group.

## Discussion

In ocular tissues, myocilin has been identified in the TM, sclera, aqueous humor, ciliary body, choroid, cornea, iris, lamina cribosa, vitreous, retina and optic nerve (Resch and Fautsch, 2009). Myocilin is mainly existed in TM cells and only causes glaucoma when mutated. Furthermore, HEK 293T, COS-1, COS-7 and ARPE-19 (from human retinal pigment epithelium) cell lines that were used alone or in combination according to previous researches to explore the general and the ocular characteristics and function of myocilin (Sanchez-Sanchez et al., 2007; Shepard et al., 2007; Aroca-Aguilar et al., 2008). It should be noted that in the TM cells (both immortalized and primary) and the cell lines mentioned above, endogenous myocilin is unable to be detected. Therefore, we performed intracellular and extracellular molecular characterization of myocilin and explored possible correlations between structural alterations and functional consequences using HEK 293T, COS-7 and iHTM cells expressing exogenous myocilin. iHTMCs were used to explore the ER localization of variant myocilins in cells, and their colocalization with autophagy marker LC3. Since the transfection efficiency of iHTMCs is not sufficient for the analysis of protein secretion and cellular oxidative stress, we selected HEK 293T and COS-7 cells that are easily transfected for our study. Compared with HEK 293T cells, COS-7 cells are more adherent and show advantages in staining experiments. Therefore, COS-7 cells were used in the study of oxidative stress.

Sanchez-Sanchez et al. (2007) characterized intracellular proteolytic cleavage of myocilin and identified calpain II as a myocilin-processing protease. The processed C-terminal domain is reportedly secreted into cell culture medium or the human aqueous humor, whereas the N-terminal fragment remains inside the cell (Aroca-Aguilar et al., 2008; Wang et al., 2019). However, Kwon et al. (2009) reported that the N-terminal fragment is also secreted into the medium. In accordance with previous studies, we detected full-length myocilin as well as cleaved C-terminal products both inside and outside cells. Nevertheless, the processed N-terminal fragments may have been mostly retained inside the cell and were probably part of the insoluble cell fraction, as they were not detected in the cell medium or soluble cell fraction. It was reported that the perfusion of the C-terminal fragment of myocilin did not influence outflow resistance of aqueous humor (Goldwich et al., 2003). However, the exact role of C-terminal fragment of myocilin in TM or other tissues remains to be clarified. Myocilin can dimerize or multimerize with itself through its leucine zipper or coiled-coil domain. Under nondenaturing conditions, the culture medium and cell lysates of cells overexpressing myocilin for 48 h demonstrated a regular size pattern of myocilin aggregates consisting of various bands larger than 130 kDa, suggesting that myocilin is prone to self-oligomerization. This oligomerization has been reported to be maintained by disulfide bonds (Martin et al., 2021). According to Aroca-Aguilar et al. (2008), coexpression of WT and mutated myocilin resulted in the same pattern of aggregates, which does not support obstruction of aqueous humor outflow due to an increase in the molecular size of myocilin aggregates. Regardless, the secreted products that result from proteolytic cleavage and self-aggregation may regulate interaction of myocilin with ECM proteins such as fibronectin, collagen VI, decorin and laminin (Ueda and Yue, 2003). Overall, changes in the ECM composition will increase the resistance of aqueous humor outflow and result in high IOP.

Secretion status of myocilin could be defined by immunoblotting or luciferase assay (Nakahara and Hulleman, 2022). Our data agreed very well with previous studies showing intracellular sequestration of the G367R, P370L, K423E, C433R, I477N myocilin mutants and secretion of the N57S, Q48H, R82C, R126W, R158Q, T293K, A445V variants (Shepard et al., 2003; Gobeil et al., 2006). Secretion property of eleven variants were first tested in this study. These were the V53A, N57D, T209N, L215Q, G326S, V329M, D380N which were secreted, and the C25R, Y371D, D380Y, S425P which were not released outside the cells. Interestingly, N-terminal variants of myocilin were secreted in addition to the C25R variant; in contrast, 69.7% (23/33) of C-terminal variations, located in the OLF domain of myocilin, resulted in secretion defects. In terms of pathogenicity, 100% of MYOC neutral polymorphisms induced normal secretion of myocilin, but 87% (20/23) of MYOC mutation-encoded myocilin caused secretion defects. Together with quantification data, we propose that the secretion defect of C25R variant is due to the dysfunction of signal peptide, causing the variant protein cannot enter ER for processing and be degraded at early stage. In line with the work of Kasetti *et al*, we found that MYOC mutations in C-terminus increased the level of insoluble myocilin (Kasetti et al., 2021). Therefore, the nonsecretion of C-terminal mutants may be caused by the decreased solubility of myocilin resulting from protein misfolding.

The crystal structure of myocilin has been illustrated by previous studies (Hill et al., 2017; Martin et al., 2021). The OLF domain is highly conserved and participates in protein‒protein interactions, which are associated with various human diseases, including inflammatory bowel disease, cancer, and glaucoma (Hill et al., 2015). Mutations in the OLF domain of myocilin promote the formation of amyloid fibrils, which are difficult to be degraded (Orwig et al., 2012; Hill et al., 2014). Donegan et al. (2015) identified three regions of myocilin-OLF that are sensitive to amyloid aggregation: Loop B-10/C-11 and cation-Π; a hydrophobic β-sheet; and Ca^2+^ site environs. In our study, mutations in Loop B-10/C-11 and cation-Π (G367R, P370L, and K423E) and the hydrophobic β-sheet (C433R and I477N) resulted in cellular myocilin accumulation. Notably, two mutations in D380 (D380N and D380Y), which are located in the Ca^2+^ site environs, showed different results. D380N mutation led to extracellular and intracellular myocilin, whereas intracellular myocilin was only detected with the D380Y variant. This may be due to the different changes in protein structure caused by these mutations. Conformational alterations, such as H-bonds and steric clashes, may influence the folding of proteins, which can further change their function or characteristics (Rose, 2021). Correlation analysis of structure-secretion-pathogenicity of variant myocilins revealed a possible role for steric clash in affecting the secretion and pathogenicity of myocilin variants. This hypothesis may explain the difference in secretion characteristics between D380N and D380Y variants, as well as G326S and G326R variants. Certainly, more research is needed to elucidate the mechanism by which the OLF domain affects the function of myocilin.

Myocilin is mainly expressed in TM cell, and excessively accumulated in ER when MYOC is mutated or overexpressed, which may trigger or disrupt protein clearance mechanisms including ER-associated degradation (ERAD) and autophagy (Kasetti et al., 2021; Tanji et al., 2021). However, previous studies only reported the difference between WT-MYOC and mutated-MYOC (Y437H, G364V, Q368X, *etc*), ignoring the alteration induced by MYOC neutral polymorphisms. In this study, two nonsecreted variants (S425P, D380Y) and four secreted variants (T293K, V329M, A445V, D380N) that have not yet been studied were chosen for further research. We found that compared to the MYOC neutral polymorphism, myocilin encoded by MYOC mutations accumulates in the ER and induces autophagy impairment. A study by Bosley et al identified a spectrum of mitochondrial dysfunction in POAG patients that is associated with cellular oxidative stress and suggests that mitochondrial abnormalities may be a risk factor for POAG (Abu-Amero et al., 2006). In addition, it is reported that mutant myocilin sensitizes cells to oxidative stress and anti-oxidative stress enzyme deficiency promoted the occurrence and degree of POAG phenotype in mouse model that carrying MYOC mutation (Joe and Tomarev, 2010; Joe et al., 2015). Consistently, we demonstrated that MYOC mutations (G367R, P370L) inhibited mitochondrial function and increased cell sensitivity to oxidative stress. Combined with previous findings, the pathogenicity of MYOC variations is associated with myocilin secretion defects, altered steric clash, ER localization, decreased autophagic activity and increased oxidative stress. The secretion disorder of myocilin may be an important determinant in the pathogenic mechanism of MYOC mutations.

According to the work of Donegan et al., the pathogenicity of four variants (T293K, V329M, S425P, A445V) were evaluated differently than original assignments based on location in myocilin-OLF structure (Donegan et al., 2015). However, based on the latest database, the clinical significance of three of these four variants (T293K, S425P, A445V) is consistent with Donegan’s prediction (Hewitt et al., 2008). Our study revealed that V329M variant, a controversial variant, exhibited no secretion defect and ER localization, as well as autophagic disruption and oxidative injury in cells. L215Q, G326S, and T377M-MYOC variations are defined as POAG-causing mutations. However, the L215Q mutation has only been reported in one case thus far, and the G326S mutation lacks specific functional analysis and clear genetic evidence, rendering the clinical significance of these two variations unclear (Hewitt et al., 2008). Moreover, no abnormal molecular or cellular biology was observed in cells expressing L215Q and G326S in this study. Consequently, we propose that V329M, L215Q and G326S variants are non-pathogenic. The secretion property of T377M-myocilin was examined by dot blot assay in a previous study, showing a weak gray band, indicating significantly reduced secretion of T377M-myocilin or a false positive band caused by other interfering factors (Gobeil et al., 2006). Therefore, we speculate that a secretion defect of MYOC variants is a prerequisite for the POAG phenotype, which is associated with altered steric clash of myocilin variants. MYOC mutations induce autophagy dysfunction through ER retention.

In summary, we found an interesting correlation between steric clash alterations and the secretion property of MYOC missense mutants. Nonsecreted myocilin is retained in the ER, inducing a series of stress responses, including impaired autophagic degradation and increased oxidative injury. Considering that the *in silico* approach has inherent limitations of not considering the global changes that can only be studied in solution, our findings need to be validated by further comprehensive experimental analysis. Nevertheless, the *in silico* approach can help to elucidate the molecular pathogenesis of POAG and pave the way for similar analyses for other diseases involving the OLF domain.

## Data Availability

The original contributions presented in the study are included in the article/Supplementary Material, further inquiries can be directed to the corresponding authors.

## Supplementary material

Supplementary Figure S1 Expression of myocilin in different cell fractions. Myocilin detected in different cell fractions was quantified by ImageJ. The nonsecreted variants mainly existed in the insoluble fraction.

Supplementary Figure S2 Alteration of H-bonds and steric clashes induced by MYOC mutations. H-bonds are drawn as green dotted lines, and clashes appear as pink dotted lines. The yellow arrow indicated the altered H-bond or steric clash.

Supplementary Figure S3 Changes in molecular surface including electrostatic potential (SEP) and structure of MYOC mutations. The molecular surface is colored according to SEP using Swiss PdB Viewer, with red‒white‒blue corresponding to acidic-neutral-basic potential. The yellow dotted circle represents the region of significant alteration of SEP and surface structure.

Supplementary Table S1 Primer sequences for myocilin cDNAs.

Supplementary Table S2 Reasons for selection of MYOC variants.

Supplementary Table S3 Structure-secretion-pathogenicity analysis of C-terminal myocilin variants.
