# Supplementary figures and images for: Structure‒function‒pathogenicity analysis of C-terminal myocilin missense variants based on experiments and 3D models
Source: Front Genet. 2022 Oct 4;13:1019208. doi: 10.3389/fgene.2022.1019208 (PMC9577182; doi:10.3389/fgene.2022.1019208)

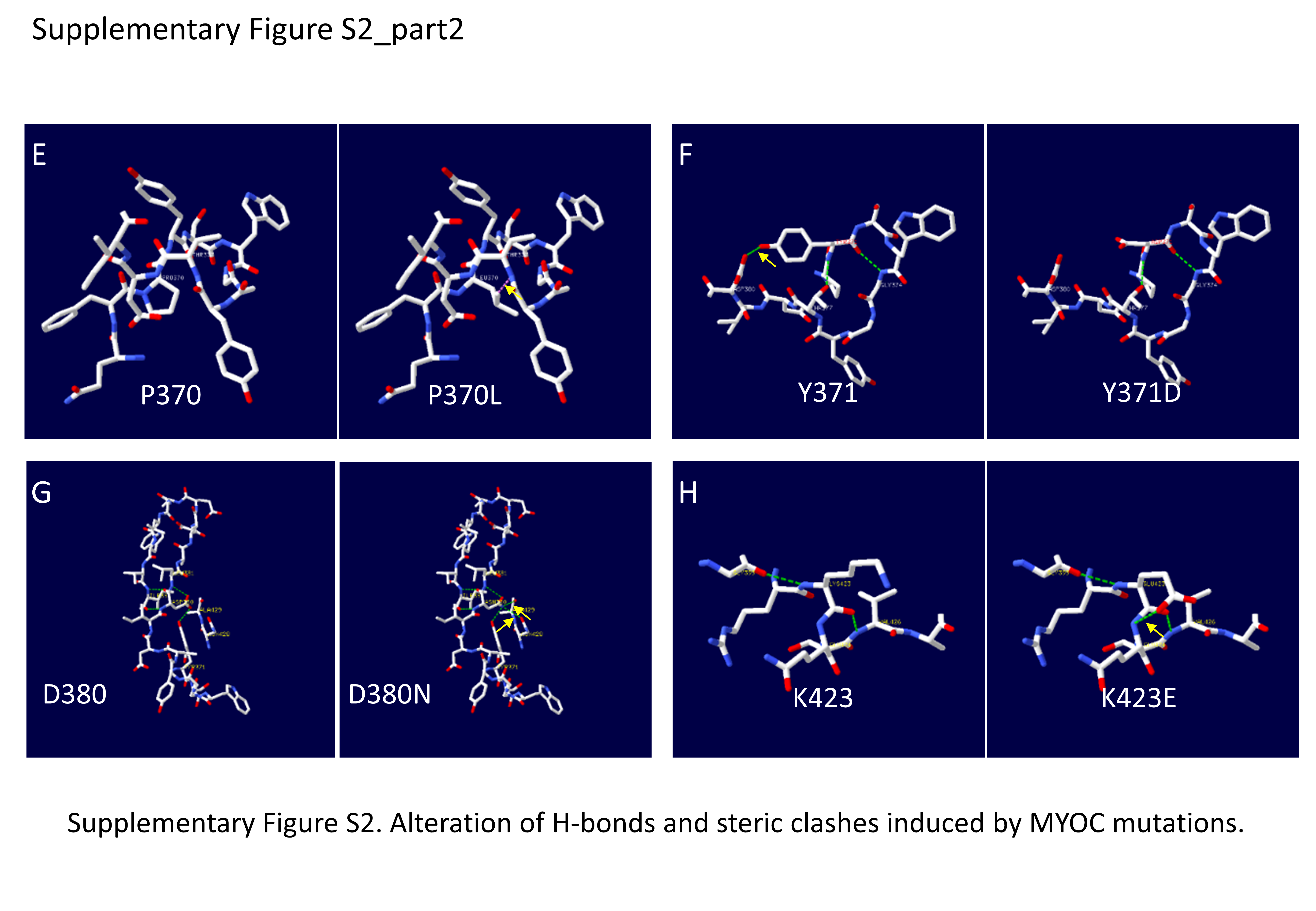

Supplement: Supplementary file 3 [file Image3.tif]

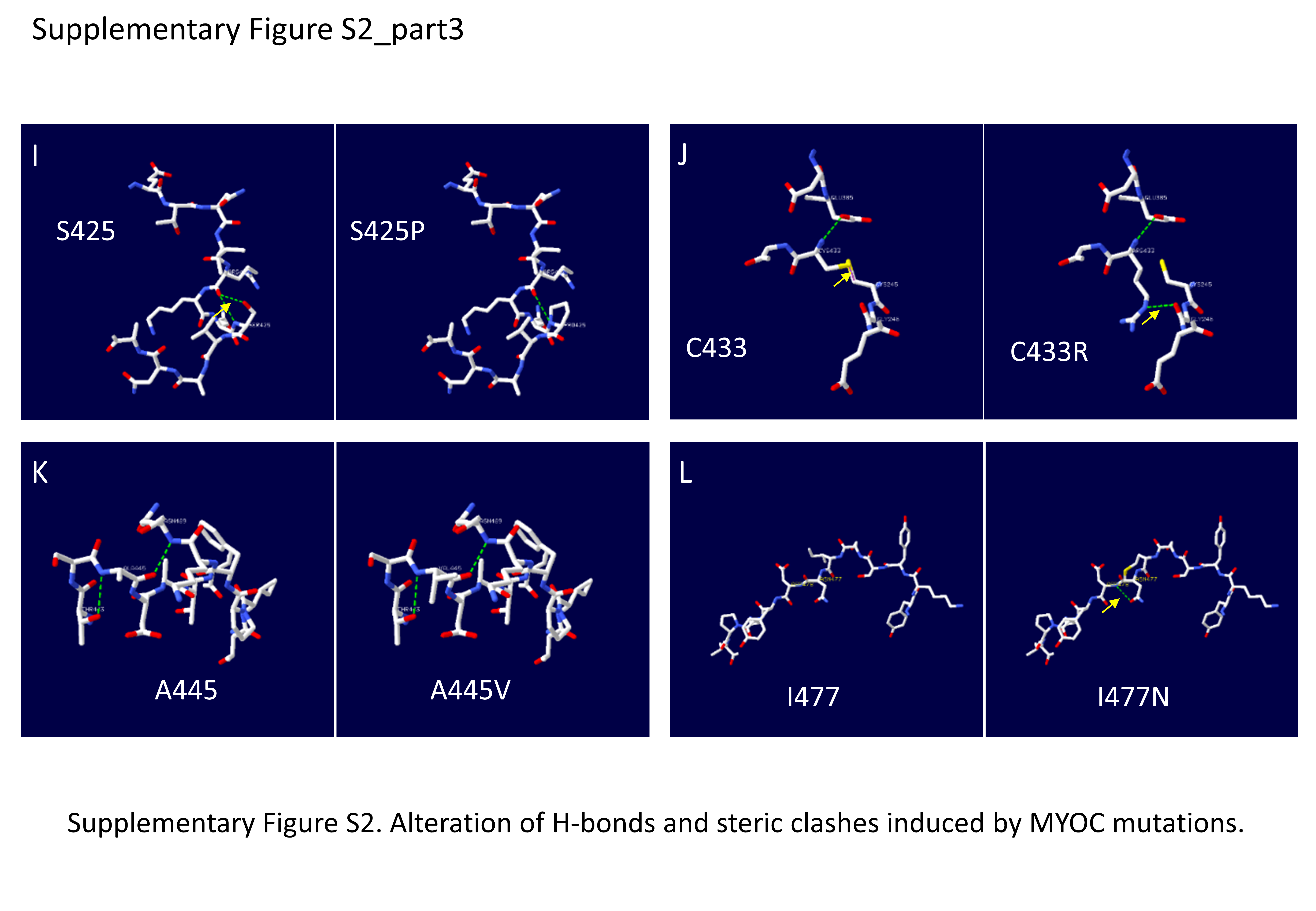

Supplement: Supplementary file 4 [file Image4.tif]

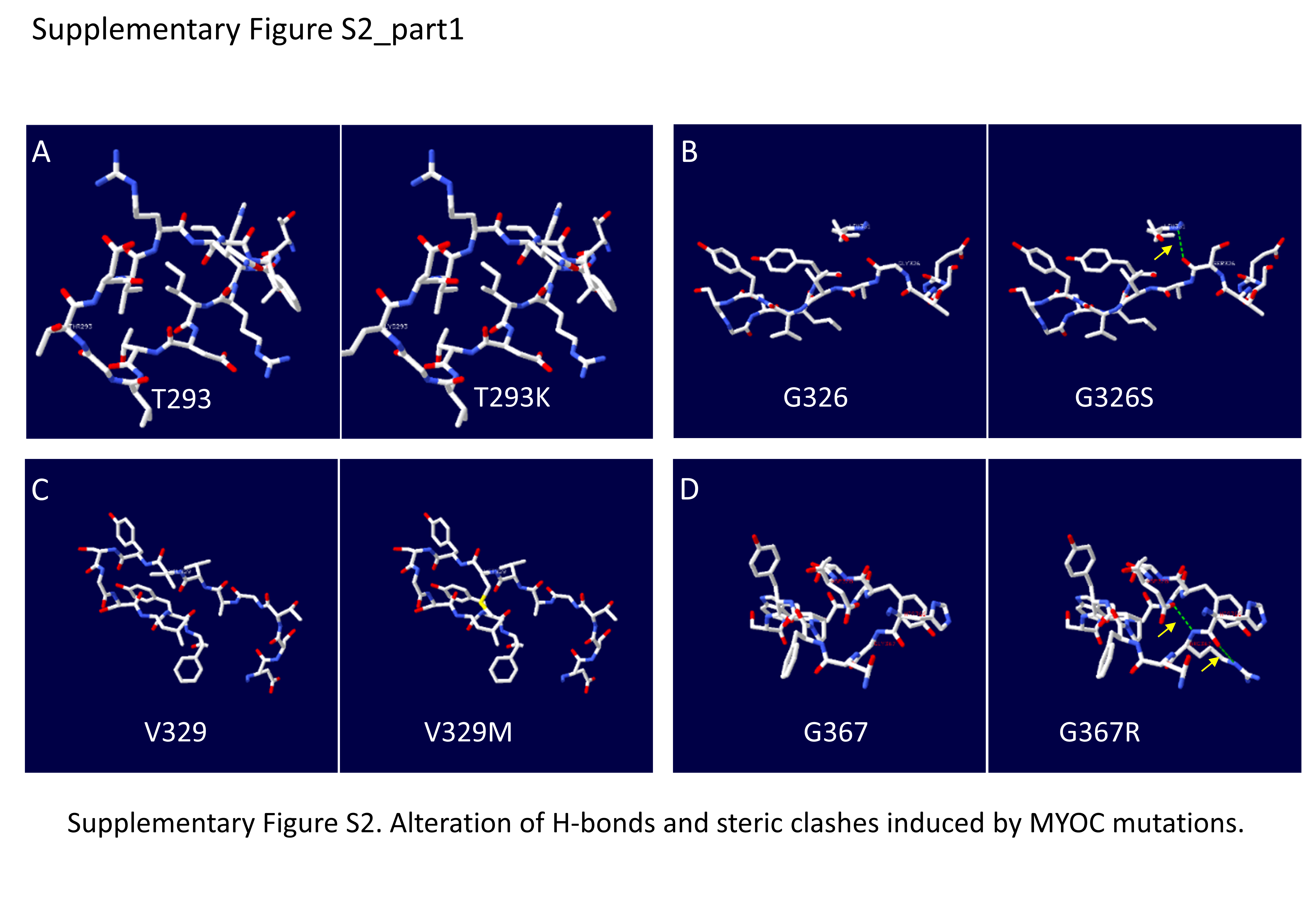

Supplement: Supplementary file 5 [file Image2.tif]

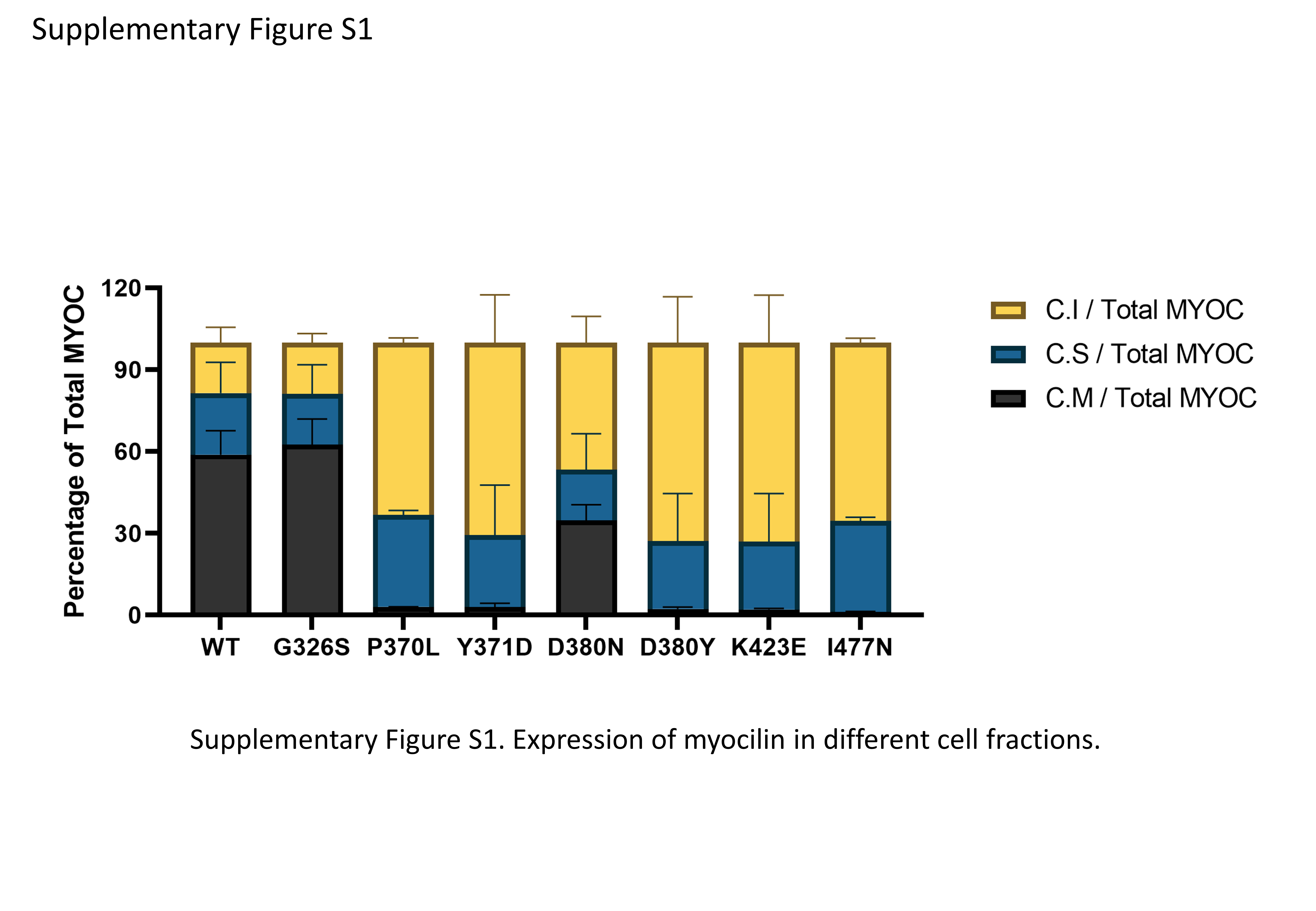

Supplement: Supplementary file 6 [file Image1.tif]

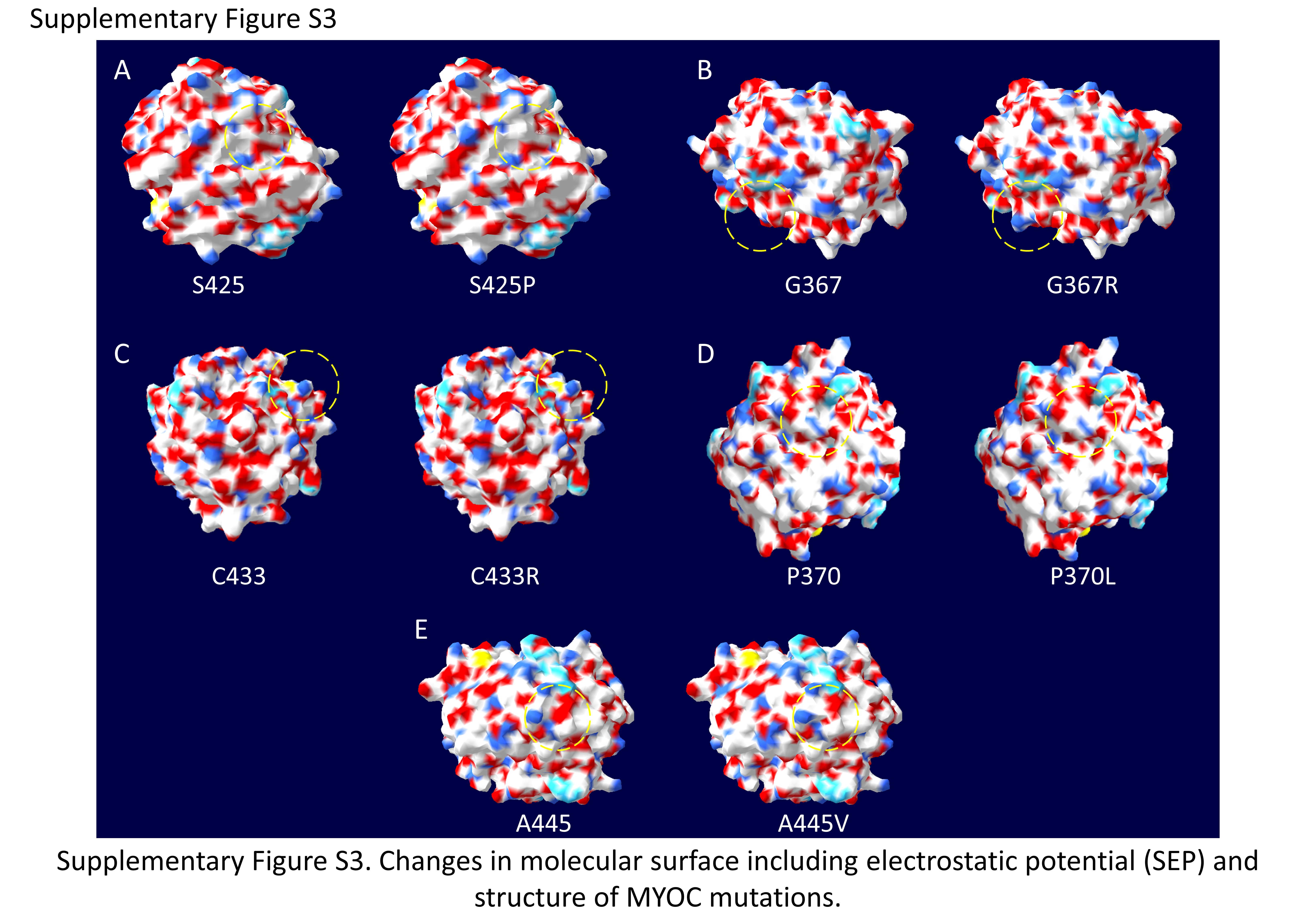

Supplement: Supplementary file 8 [file Image5.tif]
